# Supplementary material for: Expression of the Immunohistochemical Markers CK5, CD117, and EGFR in Molecular Subtypes of Breast Cancer Correlated with Prognosis
Source: Diagnostics (Basel). 2023 Jan 19;13(3):372. doi: 10.3390/diagnostics13030372 (PMC9914743; doi:10.3390/diagnostics13030372)
Supplement: Supplementary file 1 [file diagnostics-13-00372-s001.zip › diagnostics-2140128-supplementary.pdf]

Supplement

Immunohistochemical staining

All immunohistochemical stainings were performed at the Institute of Pathology of the University Hospital Erlangen.

Supplementary Table S1. Antibodies used for immunohistochemical staining

Antibody	Dilution	Clone
CK5	1:50	H587
CD117	1:100	EP10
EGFR	Ventana donor ready to use	3C6
ER	1:40	EP1
PR	1:50	PgR 636
MIB Ki-67	1:100	MIB 1
HER2	1:1000	Polyclonal

CK5 cytokeratin 5, *CD117* cluster of differentiation 117, *EGFR* epidermal growth factor receptor, *ER* estrogen receptor, *PR* progesterone receptor, *HER2* human epidermal growth factor receptor 2

For IHCs stainings, the IHC protocol was performed on a Ventana Benchmark Ultra automated platform (Ventana Medical Systems, Inc., Oro Valley, AZ, USA). 2 µm thick sections of the TMA FFPE blocks were mounted on adhesive glass slides. Antibodies used are described in Supplementary Table S1. For CK5, the antibody was applied for 60 min at 37 °C after pretreatment (64 min) with ULTRA Cell Conditioner 1 (Ventana). For CD117, the antibody was applied for 32 min at 37 °C after pretreatment (36 min) with ULTRA Cell Conditioner 1 (Ventana). For EGFR, the antibody was applied for 32 min at 36 °C after pretreatment (64 min) with ULTRA Cell Conditioner 1 (Ventana). The binding of each antibody to the corresponding antigen was visualized using the optiView DAB IHC Detection

Kit (Ventana) and, subsequently, sections were counterstained with hematoxylin and Bluing Reagent (Ventana).

Evaluation of the immunohistochemical stains

Immunohistochemical staining was evaluated with a microscope with a discussion device (Zeiss Axio Imager.A2). The stains were evaluated according to the following criteria:

Estrogen- and Progesterone receptor

Hormone receptors were evaluated according to the percentage of positive nuclei in the tumor cells and their staining intensity (weak, moderate, strong) [64]. Only cases with nuclear expression in > 1% of tumor cells were considered positive [67].

HER2

Completely negative staining was classified as score 0, weak membranous staining of more than 10% of tumor cells as score 1+, weak to moderate circular membranous staining of more than 10% of tumor cells as score 2+, and strong circular membranous staining of more than 10% of tumor cells as score 3+. All results assessed with a score of 3+ were considered positive in this analysis, whereas intensities 0 to 1+ were considered negative. For cases with a score of 2+, chromogenic in situ hybridization was performed and patients with gene amplification and a HER2/CEN17 ratio of > 2 were considered HER2 positive [68,69].

Ki-67

In a low magnification, the area with the most positively labeled tumor cells was first selected. Subsequently, the percentage of nuclearly labeled tumor cells of all tumor cells was indicated in the magnification with the 40x objective.

CK5, CD117, EGFR

Evaluation of these biomarkers is explained in detail in the manuscript.

Supplementary Table S2. Scoring of CK5, CD117, and EGFR IHC

<table>
<tr><th>IHC Score</th><th>Score definition</th><th>CK5 status</th><th>EGFR status</th><th>CD117 status</th></tr>
<tr><td>0</td><td>Completely negative staining</td><td>Negative</td><td>Negative</td><td rowspan="2">Negative</td></tr>
<tr><td>1</td><td>Weak positivity or less than 10% positive cells*</td><td rowspan="3">Positive</td><td rowspan="3">Positive</td></tr>
<tr><td>2</td><td>More than 10% positive cells and moderate staining</td><td rowspan="2">Positive</td></tr>
<tr><td>3</td><td>More than 10% positive cells and strong staining</td></tr>
</table>

* A case with 100% of the cells stained with weak intensity has to be scored as Score 1.

CK5 cytokeratin 5, *CD117* cluster of differentiation 117, *EGFR* epidermal growth factor receptor, *IHC* immunohistochemistry
